# A rare presentation of simple renal cyst: gastrointestinal obstruction 

**Published:** 2018

**Authors:** Amir Sadeghi, Mohammad Amin Shahrbaf, Hamid Asadzadeh Aghdaei, Komeil Esmaeilinejad, Mohammad Reza Zali

**Affiliations:** 1 *Gastroenterology and Liver Diseases Research Center, Research Institute for Gastroenterology and Liver Diseases, Shahid Beheshti University of Medical Sciences, Tehran, Iran*; 2 *School of Medicine, Shahid Beheshti University of Medical Sciences, Tehran, Iran*

**Keywords:** Intestinal obstruction, Renal cyst, Endoscopic ultrasound

## Abstract

Simple renal cysts are one of the most common lesions in elderly. These cysts are usually asymptomatic but when the size of these cysts increase, we would see symptoms such as hypertension, hematuria, flank pain or urinary obstruction. In this study, we explore a case of small bowel obstruction that presented with nausea, repeated vomiting that causes hematemesis, and a submucosal obstructive lesion that was seen in Esophagogastroduodenoscopy (EGD). After endoscopic ultrasound (EUS) evaluation, we detected a large simple renal cyst and approved our diagnosis with CT scan. We planned a medical treatment for this patient that consist consuming small size meals, 5 to 6 times a day, and high calorie liquids in small volumes. We conclude that simple renal cyst can be one of the cause of extrinsic intestinal obstruction and EUS is affective for differentiation of intrinsic submucosal lesion from extrinsic compression.

## Introduction

 Simple renal cysts are benign masses which are formed in the kidneys for unknown reasons and can be inherited or acquired. These cysts are present in 10% of the general population and their prevalence increase with age (1, 2). These cysts are usually unilateral and do not cause renal dysfunction or other systemic disorders so they are usually asymptomatic (3). If the size of these cysts be more than 5cm, they may cause symptoms such as flank pain, hematuria, hypertension or pelvicalyceal obstruction (4, 5). These cysts are accidentally detected in the elderly by ultrasound or CT scan (1).

Submucosal lesions are seen in 0.36% of upper gastrointestinal endoscopy cases (6-8). Gold standard method for detecting these lesions is endoscopic ultrasound (EUS) (6). These mucosal lesions include a wide range of disorder from benign to pre-malignant and malignant lesions and cause obstructive symptoms like nausea and vomiting (9, 10).

In this study, we reported a rare case of duodenal obstruction secondary to a large simple renal cyst. 

## Case presentation

An 81-year-old man presented to emergency department with the chief complaint of hematemesis which lasted for 7 days. His hematemesis started after three times none bloody emesis. He mentioned history of weight loss, recurrent epigastric pain and post prandial vomiting for one year. The vomitus occurred one to two times daily shortly after meal consumption. He had past medical history of a laparoscopic cholecystectomy five years ago, an ischemic brain stroke two years ago, and he was paraplegic due to a lumbar herniated disc about one year ago. His familial history was negative for GI disorders.

On physical examination the patient was cachectic and extremely weak with a blood pressure of 90/40 mmHg and heart rate of 110 beats/min. Respiratory rate was 22/min and oral temperature was 36.8. The sclera of patient was not icterus, lymphadenopathy, abdominal tenderness or palpable mass were not found. Some of the laboratory finding of this patient summarized in table 1.

**Table 1:** Laboratory test at the admission

Laboratory Test	Finding
WBC(µl)	6900
Hemoglobin(g/dl)	10.8
MCV(fl/red cell)	86
RDW%	14
Platelet(µl)	241000
Creatinine(mg/dl)	1.1
Na(meq/l)	134
K(meq/l)	4
AST(U/L)	28
ALT(U/L)	40
ALP(IU/L)	153
Total Bilirubin(mg/dl)	0.9
Direct Bilirubin(mg/dl)	0.2
PTT(second)	36
INR	1.2
LDH(U/L)	446
CEA(ng/ml)	1.3
ESR(mm/hour)	25
CRP(mg/dl)	56

**Figure 1:**
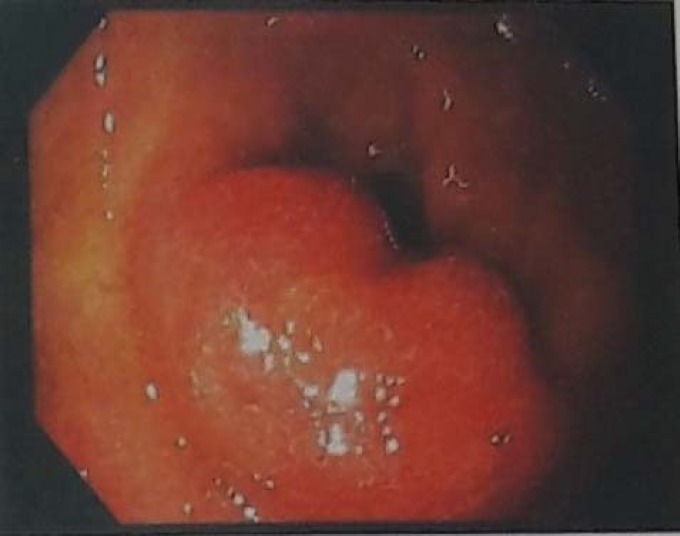
Submucosal region in the bulb of duodenum that caused partial obstruction and obstructive symptoms

Treatment initiated by infusion of crystalloid fluids and intravenous pantoprazole. After the supportive care, esophagogastroduodenoscopy (EGD) was performed. EGD revealed a linear clean base ulcer in lower third of esophagus (Mallory Weiss syndrome), that has been caused by repeated vomiting, and also a Submucosal lesion which was observed in bulb area of duodenum (Figure 1). 

**Figure 2:**
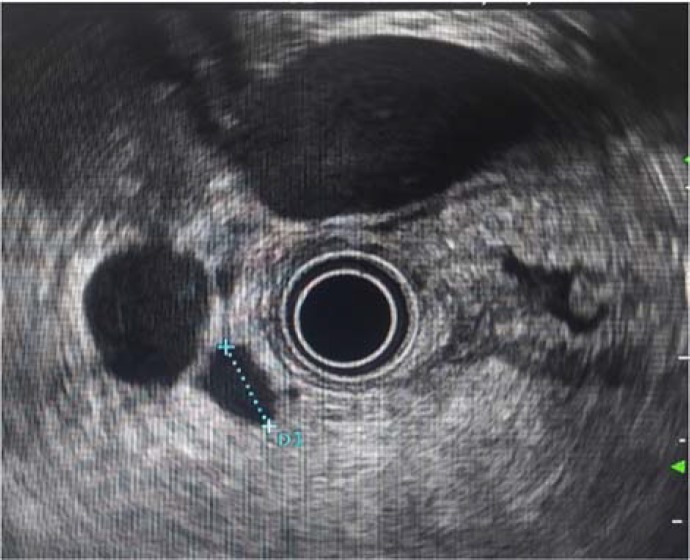
An anechoic lesion that been related to duodenal wall, without disturbing the integrity of wall. The location of the lesion was seemed in right kidney

Endoscopic ultrasonography was subsequently performed and demonstrated one anechoic lesion measuring 45×55 mm that adjacent to duodenal wall although integrity of the duodenal wall layers was intact. The most possible location of the lesion was right kidney (Figure 2). 

**Figure 3:**
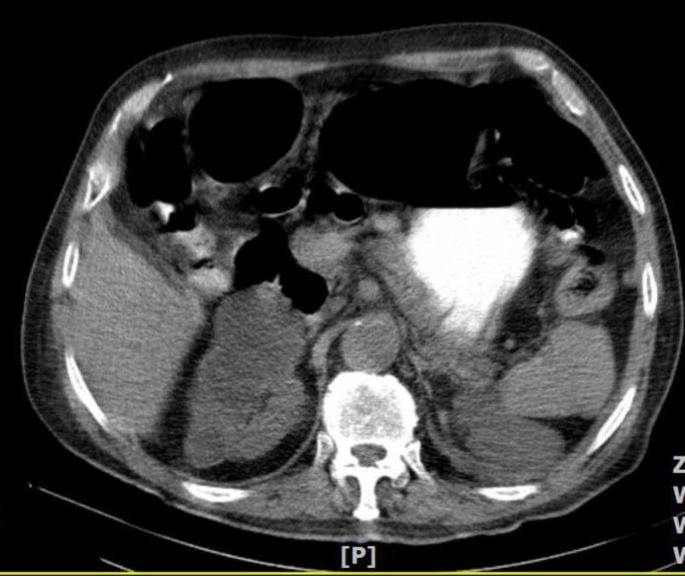
Multiple cortical cysts in both kidneys. One of the large cyst that been located in right kidney pressured on duodenum and causes partial obstruction

Unenhanced and contrast-enhanced multi slice CT scan of the abdomen and pelvic were done. In the CT scan of abdomen and pelvic region, some evidence of multiple cortical cysts was seen in both kidney and one of these cysts with the size of 45×55 mm, pressured on duodenum (Figure 3).

Conservative treatment was preferred according to the recent investigations (11), patient's adverse condition, and also patient’s refusal to surgical treatment. We started the treatment with para enteric infusion of amino acids and intralipids, infusion of crystalloid fluids and intravenous metoclopramide. The patient was advised to consume small size of meals, 5 to 6 times a day, and high calorie liquids in small volumes. The treatment plan was relatively successful and general condition of the patient improved during the next two weeks. The mentioned diet was tolerated as well by the patient.

## Discussion

This study reports a complication of simple renal cyst that presented with gastrointestinal obstruction which rarely been described in the Medline, Scopus and ISI Web of Knowledge database. Some case reports describe GI obstruction by multiple renal cyst in ADPKD (11, 12) and large renal cyst that presented with GI obstruction and pancreatitis (13). But our case is a simple renal cyst with the presentation of GI obstruction and upper GI bleeding.

Sub mucosal lesions can be located from mucosa to serosa, so it is better to named them as sub epithelial lesions (8). Compression of some structures outside of the intestinal wall can be observed in EGD as a submucosal lesion (6). Differentiating between intramural lesions and extrinsic compression may be not possible by endoscopy alone, and we can use EUS for evaluation of these sub mucosal lesions (14). Integrity of intestinal wall layer and the correlation of adjacent organs and the Sub-epithelial lesion could be observed during EUS (6, 15). 

Obstruction of intestine and specially duodenum could happen due to extrinsic compressions resulted from variety of lesions such as superior mesenteric artery (Wilkie’s syndrome), Pancreatic Adeno-squamous Carcinoma, Aortic aneurysm, and polycystic kidney disease such as ADPKD (11, 16-18). In current case, compression effect of the renal cyst on duodenal bulb lead to obstructive symptoms including feeding intolerance, vomiting, repeated retching and upper gastrointestinal bleeding (Mallory Weiss syndrome).

Since the presentation of a simple renal cyst as a cause of upper gastrointestinal obstruction is very rare, the management of such situations is unclear. In extra-luminal duodenal compression that caused by renal cysts authors have recommended conservative treatment and if the patient’s symptoms were not improved surgical intervention may be considered (11, 13). According to the mentioned articles and poor prognosis of this patient, medical treatment consisting of frequent small meals and high calorie of nutritious liquids were preferred.

Submucosal lesion could be caused by extra luminal compression and causing GI obstruction symptoms. Simple renal cysts should be one of the differential diagnosis of GI obstruction that caused by extrinsic factor.

EUS is an affective modality for detecting any submucosal lesion. If the lesion caused by an extra luminal factor, we can use CT for further inspections.

It is better to use medical treatment in extra-luminal duodenal compression that caused by renal cyst as the initial and principle approach.

## Conflict of interests

The authors declare that they have no conflict of interest.
